# The rodent endovascular puncture model of subarachnoid hemorrhage: mechanisms of brain damage and therapeutic strategies

**DOI:** 10.1186/1742-2094-11-2

**Published:** 2014-01-03

**Authors:** Elke Kooijman, Cora H Nijboer, Cindy TJ van Velthoven, Annemieke Kavelaars, Jozef Kesecioglu, Cobi J Heijnen

**Affiliations:** 1 Department of Intensive Care Medicine, University Medical Center Utrecht, Utrecht, The Netherlands; 2 Laboratory of Neuroimmunology and Developmental Origins of Disease, University Medical Center Utrecht, Utrecht, The Netherlands; 3 Division of Internal Medicine, Department of Symptom Research, The University of Texas MD Anderson Cancer Center, Houston, TX, USA

**Keywords:** Cell death, Endovascular puncture, Inflammation, Neuroregeneration, Stem cells, Subarachnoid hemorrhage, Treatment options

## Abstract

Subarachnoid hemorrhage (SAH) represents a considerable health problem. To date, limited therapeutic options are available. In order to develop effective therapeutic strategies for SAH, the mechanisms involved in SAH brain damage should be fully explored. Here we review the mechanisms of SAH brain damage induced by the experimental endovascular puncture model. We have included a description of similarities and distinctions between experimental SAH in animals and human SAH pathology. Moreover, several novel treatment options to diminish SAH brain damage are discussed.

SAH is accompanied by cerebral inflammation as demonstrated by an influx of inflammatory cells into the cerebral parenchyma, upregulation of inflammatory transcriptional pathways and increased expression of cytokines and chemokines. Additionally, various cell death pathways including cerebral apoptosis, necrosis, necroptosis and autophagy are involved in neuronal damage caused by SAH.

Treatment strategies aiming at inhibition of inflammatory or cell death pathways demonstrate the importance of these mechanisms for survival after experimental SAH. Moreover, neuroregenerative therapies using stem cells are discussed as a possible strategy to repair the brain after SAH since this therapy may extend the window of treatment considerably. We propose the endovascular puncture model as a suitable animal model which resembles the human pathology of SAH and which could be applied to investigate novel therapeutic therapies to combat this debilitating insult.

## Introduction

Subarachnoid hemorrhage (SAH) is a pathological condition in which arterial blood flows into the subarachnoid space of the brain and which is mostly caused by a ruptured aneurysm. SAH occurs at a relatively young age and has an unpredictable onset. In Western society, the incidence of SAH is 6 to 7 per 100,000 persons per year and half of the patients are under the age of 55 [1-4]. SAH is associated with a high mortality rate of 50% [1,4]. In surviving patients the insult has major consequences for quality of life. In 20 to 40% of the patients who survive the first insult, a second phase of brain damage occurs which is characterized by delayed cerebral ischemia (DCI) and is associated with increased morbidity and mortality [5-7]. DCI, also referred to as delayed ischemic neurological deficit, is a poorly understood complication of SAH in patients.

Directly after SAH, an immediate increase in both mean arterial blood pressure (MABP) and intracranial pressure (ICP) is observed. Cerebral blood flow (CBF), oxygen tension, and cerebral perfusion pressure (CPP) decrease dramatically after SAH [8-13]. These hemodynamic changes eventually contribute to initiation of cerebral inflammation and apoptotic and necrotic cell death. Cerebral vasospasm is thought to play an important role in the etiology of DCI. It has been hypothesized that several processes occurring as a result of SAH, like inflammation, oxidative stress and hemoglobin reacting with nitric oxide (NO), initiate vasospasm leading to DCI [14]. However, this hypothesis has been increasingly questioned over the last couple of years, because vasospasm does not necessarily lead to DCI and DCI can occur in the absence of vasospasm [6].

There are only limited therapeutic options available to diminish brain injury after SAH and unfortunately, treatments that are available have a short therapeutic window. In order to design new effective treatment strategies for SAH, the pathophysiology of SAH brain damage should be studied extensively. The latter necessitates application of animal models closely resembling human pathology.

### Animal models for SAH

Several animal models of SAH are available, of which the single-hemorrhage model, the double-hemorrhage model and the endovascular puncture model are used most commonly. In the single-hemorrhage model, a standard amount (an average of 300 μl) of fresh syngeneic arterial blood is injected into the cisterna magna [15]. In the double-hemorrhage model, two injections with autologous arterial blood are given, in most cases 48 hours apart [15]. In the endovascular puncture, a suture is placed in the external carotid artery (ECA) and threaded through the internal carotid artery up to the middle cerebral artery (MCA) where the vessel is punctured. The suture is immediately retracted to limit an ischemic period in order to mimic the clinical situation as closely as possible. The endovascular puncture model is mainly performed in rats, although there are a few studies that describe the endovascular puncture model in mice. The single- and double-hemorrhage model is performed in dogs, rabbits, rats and mice. The mortality rate in the single- and double-hemorrhage experimental models is low (0 to 20%) and the models are fairly reproducible because a fixed amount of blood is injected into the subarachnoid space [10]. In contrast, the mortality of the endovascular puncture model is higher (approximately 35 to 50%), as in humans. A larger variation in severity of outcome is also observed, as the extent of the hemorrhage itself is variable after puncture [6,10,12,16,17]. We would like to suggest that the endovascular puncture model resembles human pathology, as an increase in mean arterial blood pressure and intracranial pressure are observed in both humans and animals after SAH. In the endovascular puncture model in rats, magnetic resonance imaging (MRI) showed a modest constriction of blood vessels two days after SAH, which might mimic vasospasms observed in human SAH pathology [18]. In this review, our main focus is on the endovascular puncture experimental model.

### Aim of this review

The aim of this review is to give an overview of *novel* therapeutic approaches to prevent or treat the pathological consequences of SAH. Several other experimental therapies have been described earlier to combat brain injury. This review aims at describing a selection of anti-inflammatory, neuroprotective and neuroregenerative pathways in the endovascular puncture model. We will also focus on diagnostic and mechanistic similarities and distinctions between the endovascular puncture model in animals and the human pathology of SAH.

### Current therapies

The few therapies currently available for SAH focus on prevention of re-bleeding and prophylaxis for vasospasm [19-21]. Occlusion of the aneurysm that causes SAH is usually sought surgically by clipping or by endovascular coiling. In 5 to 10% of cases, endovascular coiling is not possible due to morphological characteristics or location of the aneurysm. However, endovascular coiling is minimally invasive and therefore, it is preferred over traditional neurosurgical clipping [20,21]. Hypothermia has been shown to reduce edema formation and intracranial pressure after SAH and could be an important means to control fever in SAH. However, hypothermia has also been associated with shivering in SAH patients which is negatively related to outcome after SAH [22,23]. A more invasive treatment that is sometimes applied is decompressive craniectomy (DC) aimed at reducing intracranial pressure after SAH. However, there is continuing debate about the timing and laterality of DC and on the long-term consequences [24].

Nimodipine, a calcium channel blocker, is used as a prophylaxis for vasospasm and treatment with nimodipine is started for all patients at admission. However, the exact mechanism of the beneficial effect of nimodipine is unknown [21]. In addition, treatment with magnesium sulphate has been associated with a reduction in DCI, leading to an improvement of clinical outcome. However, these positive effects of magnesium sulphate have not been confirmed in all studies [25,26]. The symptomatic treatment of vasospasm consists of triple H therapy (hypertension, hypervolemia, and hemodilution) with the goal of increasing CBF, CPP and to improve blood flow and oxygen delivery to the cerebral circulation. Although triple H therapy improves CBF and CPP, it has not been proven to reduce the incidence of DCI and mortality [19,21].

### Cerebral inflammation after SAH and possible therapeutic strategies

#### Influx of immune cells

Inflammation is invariably associated with brain insults and has been suggested to be a major contributor to brain damage after SAH. Increased levels of cytokines in plasma and cerebrospinal fluid (CSF) have been shown after SAH [27,28]. In rodents, SAH induces a systemic increase in pro-inflammatory cytokines and chemokines [29]. Inflammatory cells like granulocytes and macrophages are attracted to the injured brain by cerebral production of chemoattractants and expression of adhesion molecules like integrins and selectins on endothelial cells, leukocytes, microglia, and neurons [30,31]. In SAH patients, intercellular and vascular adhesion molecules-1 (ICAM-1 and VCAM-1) have been shown to be upregulated in both CSF and serum [32,33].

Moreover, increased blood brain barrier (BBB) permeability after SAH favors influx of peripheral immune cells into the brain [34]. Matrix metalloproteinase 9 (MMP9) has been shown to be increased after SAH in the endovascular puncture model [35]. MMP9 is a collagenase that facilitates transport of immune cells to site of injury by degrading tight junctions and basal membrane proteins of the extracellular matrix (ECM) [36].

Macrophages and neutrophils enter the subarachnoid space early after SAH (within hours) where they activate resident microglia, astrocytes and neurons [37,38]. Neutrophils are primarily engaged in phagocytosis of red blood cells in the subarachnoid space after which they degranulate and die [37]. Neutrophils are also detected in the CSF of SAH patients [39]. Migration to the lesion site and activation of macrophages/microglia seems to be an ongoing inflammatory process after SAH; our data show that active macrophages/microglia can still be detected even up to three weeks post-SAH in the endovascular puncture model (Kooijman, *et al*., unpublished data) [38,40]. T-cells also enter the damaged area after SAH but only two days after the SAH [38].

#### Cytokine production

Activated immune cells secrete cytokines, chemokines, and reactive oxygen species (ROS). Several cytokines in the systemic circulation and/or CSF have been reported to increase after SAH in humans [41-43].

Tumor necrosis factor (TNF)-α is one of the first cytokines that increases after SAH in the endovascular puncture model. TNF-α regulates various processes: the expression of other pro-inflammatory cytokines, immune cell function and trafficking, and initiation of cell death [44-46]. In the endovascular puncture model, TNF-α expression has been detected in the cortex after SAH [40,47]. An increase in TNF-α protein has also been demonstrated in plasma and CSF of SAH patients [44-46,48]. Moreover, inhibition of TNF-α via an anti-TNF-α antibody decreases vasospasm and reduces expression of pro-apoptotic proteins in the single-hemorrhage model [49,50]. In line with this, it is interesting to mention that a phase I study for the use of the TNF-α antagonist etanercept in SAH patients has been started [http://clinicaltrials.gov/show/NCT01865630].

**Figure 1:**
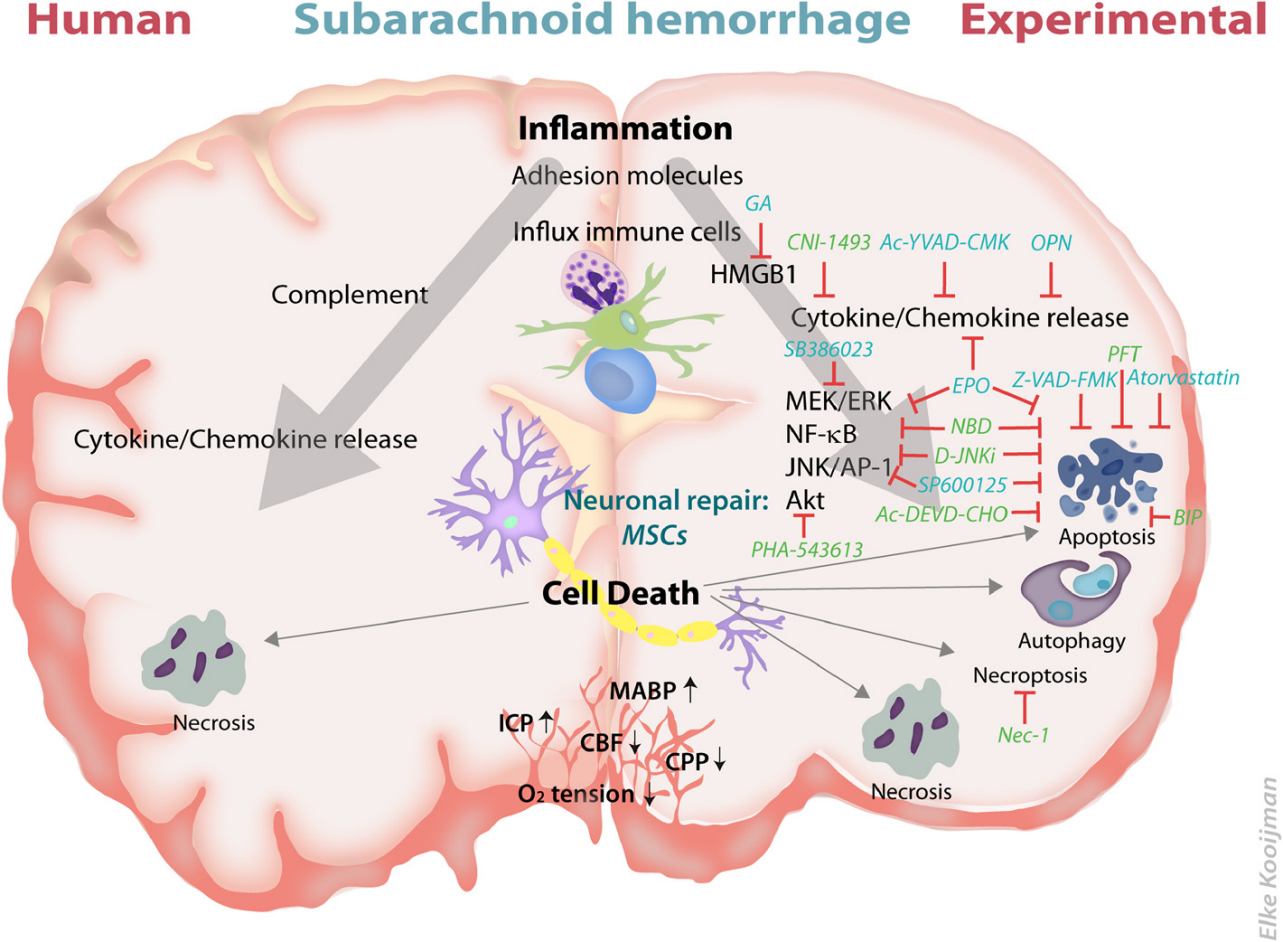
**Therapeutic targets and mechanisms of brain damage in human and experimental subarachnoid hemorrhage (SAH).** Schematic overview of known brain damage mechanisms in humans (left side of the figure) and experimental animals models (right side of the figure). Possible therapeutic options which have been explored in the endovascular puncture model are depicted in light blue. Suggested novel therapeutic options strategies are depicted in green. The red lines depict the target of inhibition. From top to bottom: SAH induces inflammation in the brain reflected by increased expression of adhesion molecules, increased influx of immune cells (depicted are neutrophils, macrophages and T-cells), increased activity of complement system, high mobility group box-1 (HMGB-1) expression, increased expression of transcription factors and cytokine/chemokine production. Different forms of neuronal cell death after SAH are observed; that is, apoptosis, autophagy, necroptosis and necrosis. Lower section: physiological factors in the brain which are altered after SAH. Abbreviations: BIP, Bax-inhibiting peptide; CBF, cerebral blood flow; CPP, cerebral perfusion pressure; EPO, erythropoietin; GA, glycyrrhizic acid; ICP, intracranial pressure; MABP, mean arterial blood pressure; MSCs, mesenchymal stem cells; NBD, NEMO binding domain peptide; Nec-1, necrostatin-1; OPN, osteopontin; PFT, pifithrin.

Interleukin (IL)-6 is secreted as an acute phase protein by many cell types including macrophages, endothelial cells, and activated microglia [51,52]. Recently, it has been shown that IL-6 also stimulates ROS production in the brain [53]. In the rat endovascular puncture model, IL-6 is expressed early after SAH [47,54]. To the best of our knowledge, no specific IL-6 inhibitor has been used in experimental SAH models. However, the cytokine inhibitor CNI-1493 (semapimod) inhibits IL-6 and decreases vasospasm in an experimental vasospasm model [55]. Therefore, CNI-1493 could be a suitable option to investigate the importance of IL-6 in SAH (see Figure 1).

IL-1β has many functions including induction of pro-inflammatory cytokines, attraction of leukocytes to the site of damage and activation of immune cells. There are contradicting results regarding whether IL-1β expression is increased in CSF and plasma of SAH patients; some studies claim no change in IL-1β after SAH [42,56] while others observed that IL-1β is increased five to nine days after SAH [41,57]. In the endovascular puncture model, expression of the precursor- and mature-form of IL-1β are elevated in the brain after SAH [35,54,58]. Greenhalgh *et al*. (2012) showed that next to IL-1β expression, IL-1α expression is increased in the brain after SAH [59].

The detrimental role of IL-1β in SAH brain damage has been demonstrated by Sozen *et al*. (2009) who showed that Ac-YVAD-CMK, a caspase-1 inhibitor, reduces mortality after SAH (see Figure 1) [35]. As caspase-1 proteolytically cleaves the precursor form of IL-1β, Ac-YVAD-CMK inhibits IL-1β maturation [35,60].

#### Molecular signaling pathways

Mitogen activated protein (MAP) kinases are important signaling pathways that are activated by a variety of stress stimuli and are involved in, for example, regulation of cytokine/chemokine expression. Activation of the MAP kinases extracellular signal-regulated kinases (ERK) and p38 have both been reported after experimental SAH [35,54,61,62]. Inhibition of the MEK/ERK pathway by the Raf inhibitor SB386023-b has been shown to reduce expression of MMP9 and pro-inflammatory cytokines IL-1β and IL-6 after SAH [62]. In line with this study, inhibition of the p38 pathway by SB203580 has been shown to reduce vasospasm, reduce TNF-α expression in CSF, reduce brain edema and improve behavior after experimental SAH [60,63].

### Cell death mechanisms after SAH and possibilities for neuroprotection

#### Apoptosis

Apoptosis is the programmed form of cell death characterized by cell shrinkage, nuclear condensation and DNA fragmentation, membrane blebbing and loss of adhesion [64]. During apoptosis, activation of the caspase cascade is a key phenomenon, however, caspase-independent apoptosis can also be operative [65].

There are numerous reports showing activation of the extrinsic (death-receptor activated) and intrinsic (mitochondria-activated) routes of apoptosis after SAH in the endovascular puncture model but their relative contribution is still unclear. Several studies show increased terminal deoxynucleotidyl transferase dUTP nick end labeling (TUNEL) staining, significant increases in caspase-3, caspase-8, cytosolic p53 and cytosolic cytochrome *c* expression in the brain after SAH in the endovascular puncture model [66,67]. Possibly the severity of SAH directs the predominant use of the intrinsic or extrinsic route. Besides elevated levels of pro-apoptotic markers, DNA fragmentation was also shown to be increased in SAH rats [67,68]. In contrast, Thal *et al*. (2009) did not observe neuronal cell death after SAH [12].

There are several molecules within the apoptotic cascade that could be targeted for neuroprotection after SAH, for example, active caspases, p53, the mitochondrial membrane and death receptors [69,70]. We will describe a selection of strategies aimed at reducing apoptosis at various levels of the cascade below.

A decrease in apoptotic cell death, including decreased TUNEL staining, caspase-8 and caspase-3 expression after experimental SAH has been detected after pre-treatment with the statin atorvastatin (see Figure 1) [67]. Statins are already Food and Drug Administration (FDA)-approved drugs used by patients to lower cholesterol and are also known to have anti-inflammatory and anti-apoptotic actions [67]. Atorvastatin has been used in the rat endovascular puncture model using oral administration for 15 days prior to the insult. Although promising, only the effects of pre-treatment with atorvastatin have been studied, so it is not clear whether *post*-SAH treatment with atorvastatin could also lead to neuroprotection. If post-treatment with atorvastatin would not convey protection of cerebral tissue after SAH, the clinical implication would of course be minimal.

A promising target within the apoptotic cascade may be represented by p53, a tumor suppressor protein orchestrating apoptosis at multiple levels. In the nucleus, p53 acts as a transcription factor of pro-apoptotic proteins [71]. Treatment with pifithrin (PFT)-α, an inhibitor of transcriptional activity of p53, resulted in reduction of BBB permeability, decreased neuronal apoptosis and a decreased mortality rate in the endovascular puncture model of SAH [72-74]. More recently, a non-transcriptional role of p53 has been described depending on the association of p53 with mitochondria [75]. We have shown that PFT-μ, a specific small molecule inhibitor of the mitochondrial association of p53 with mitochondria, is potently neuroprotective and has greater neuroprotective potential than PFT-α treatment in a neonatal brain damage model [71]. PFT-μ treatment strongly decreased mitochondrial p53 levels and thereby significantly inhibited mitochondrial damage, caspase-3 activation and apoptotic cell death after brain damage [71]. In contrast however, recent studies have shown that PFT-μ inhibits Heat Shock Protein 70 (HSP70) independently of p53 in tumor cells [76]. HSP70 is expressed after cellular stressors like nutrition deprivation and oxidative stress, and cerebral expression of HSP70 has been shown to be upregulated in the endovascular puncture model from day one to day five after SAH and after neonatal ischemic brain injury [77,78]. Inhibition of HSP70 in leukemic cells from acute myeloid and acute lymphocytic leukemia patients has been shown to increase apoptosis and cell cycle arrest [79,80]. Moreover, Sekihara *et al*. (2013) have demonstrated that PFT-μ increases the anti-tumor effects of hyperthermia via inhibition of HSP70 by promoting a cell cycle arrest [81]. One explanation for this apparent discrepancy is that PFT-μ may interfere via inhibition of HSP70 and by inducing a cell cycle arrest only in actively cycling tumor cells. Adult neurons are non-dividing cells, an intracellular state which may divert the mechanistic action of PFT-μ to prevention of apoptosis. In conclusion, the use of PFT-μ for SAH brain injury seems promising by its capacity to inhibit neuronal cell death most probably via prevention of p53 accumulation at the mitochondria.

Pro- and anti-apoptotic members of the Bcl-2 family regulate Bax pore formation within the mitochondrial outer membrane, which is an early key step in setting of the apoptotic cascade. Moreover, mitochondria are a source of ROS production under cellular stress conditions. Several studies have shown that ROS production plays a role in acute brain injury and vasospasm after SAH and mitochondrial respiration and activity have been shown to be disrupted after experimental SAH [82]. Interestingly, in several studies using the endovascular puncture model, an increase in mitochondrial Bax expression and other pro-apoptotic Bcl-2 family members, like PUMA, with a concomitant release of cytochrome *c* from the mitochondria, was observed after SAH indicating that this route is operative during SAH-induced brain injury [83-85]. To the best of our knowledge, to date there are no studies that directly target Bax pore formation or aim at preservation of mitochondrial integrity after SAH, whereas this could be a potent upstream therapeutic target. Wang *et al*. (2010) have shown in a model of neonatal ischemic brain injury that a Bax-inhibiting peptide (BIP) potently reduced gray- and white matter damage, improved motor and cognitive behavior, reduced mitochondrial membrane permeability and subsequent caspase activation [86]. Together these data indicate that the use of BIP could be a serious option for treating SAH-induced brain injury.

Direct inhibition of caspases is an alternative possibility to inhibit cerebral apoptosis after SAH. Studies by Zhou *et al*., (2004) and Iseda *et al*., (2007) have shown that the caspase-3 inhibitor Ac-DEVD-CHO and the broad caspase inhibitor Z-VAD-FMK reduce apoptosis in endothelial cells and thereby exert an anti-vasospastic effect in the blood-injection models of SAH in dogs and rabbits [87,88]. Park *et al*. (2004) has shown in the endovascular puncture model that treatment with Z-VAD-FMK reduces caspase activity, BBB permeability, vasospasm, and edema formation in the brain after SAH and improves functional outcome in rats [89].

A prominent pro-survival pathway is the serine-threonine kinase Akt. Endo *et al*. (2006) showed that phosphorylation of Akt was significantly increased in the endovascular puncture model after SAH. Inhibition of Akt signaling increased cell damage and apoptotic cell death after SAH [68]. P-Akt inactivates GSK3β, and P-GSK3β has been shown to be increased after experimental SAH [68]. We propose inhibition of GSK3β as a realistic therapeutic option for SAH. Several GSK3β inhibitors are currently being explored, like lithium and PHA-543613 which is a selective α7nAChR agonist, activating the PI3-K/Akt pathway and thereby inhibiting GSK3β. PHA-543613 has been shown to reduce BBB permeability and increased sensorimotor function after intracerebral hemorrhage [90].

#### Necrosis, autophagy and necroptosis

Necrosis is another important form of cell death in the brain. Necrosis involves unregulated digestion of cell components, cell swelling, organelle dysfunction and cell lysis, thereby releasing cellular contents like lysosomes in the extracellular environment which may promote inflammation [64,91].

High mobility group box 1 (HMGB1) is a non-histone DNA-binding protein involved in diverse intracellular functions such as inflammation and has been described as being released from necrotic cells [92-94]. Interestingly, an increase in CSF HMGB1 has been shown in SAH patients [46]. In line, Murakami *et al*. (2011) showed an increase in HMGB1 expression in a rabbit SAH model using blood injection [95]. HMGB1 released during cell death may in its turn activate cerebral inflammation by interacting with the Receptor for Advanced Glycation End Products (RAGE) or by activating Toll like receptors (TLR) like TLR2 and TLR4 on microglia and neurons [96]. Apart from a direct interaction of HMGB1 with its receptors on the cell, the effects of HMGB1 can be potentiated by forming stable complexes with endotoxins, thereby enhancing the potential of HMGB1 to induce a pro-inflammatory milieu. Moreover, HMGB1 has been shown to be capable of enhancing the cleavage of pro-IL-1β to the mature, active form by activating caspase-1 especially in a disease relevant condition such as acidosis [97]. In this respect, it is of interest that a HMGB1 inhibitor, glycyrrhizic acid (GA), decreases inflammation in several models of brain damage which may imply that inhibiting HMGB1 action in SAH might be efficacious to prevent inflammation and subsequent neuronal damage [98].

Autophagy, which involves secretion of damaged organelles by cells such as macrophages/microglia [99] can either initiate cell death or be pro-survival [99]. Several studies showed an early upregulation of autophagy markers after SAH [100-102]. The positive or negative contribution of autophagy to brain damage observed after SAH is still unknown.

Recently, another mechanism of cell death has been described named necroptosis, which is a caspase-independent programmed form of cellular necrosis sharing characteristics of necrosis, apoptosis and autophagy [103]. It has been shown that necroptosis contributes to brain pathologies like traumatic brain injury, intracerebral hemorrhage and neonatal hypoxia-ischemia. Interestingly, in these models of brain injury, it has been shown that necrostatin-1, a potent inhibitor of necroptosis, reduced brain damage, improved behavior, reduced ROS production, reduced inflammation, preserved mitochondrial function, reduced caspase-3 activation and reduced markers of autophagy like LC3-II and Beclin [104-107]. Taken together, targeting necroptosis seems a promising strategy after SAH as it may possibly allow the inhibition of multiple routes of cell death simultaneously.

### Molecular targets involved in both inflammation and cell death: possibilities for neuroprotection

#### JNK/AP-1 pathway

Inflammation and cell death are closely intertwined as inflammation often results in cell death and *vice versa*. One signaling pathway crucial for regulating both apoptosis and inflammation is the c-Jun N-terminal kinase (JNK)/activating protein-1 (AP-1) pathway. The JNK/AP-1 pathway is activated by a variety of cellular stress stimuli. The AP-1 transcription factor, when activated by JNK, regulates the upregulation of several pro-inflammatory target genes, like several cytokines and chemokines, as well as pro-apoptotic target genes, like Fas [108]. Moreover, JNK has been shown to phosphorylate several key players within the apoptotic cascade, like p53, PARP-1, Bcl-2 and Bcl-xL and Bim, which facilitates the induction of apoptosis.

Phosphorylation of JNK, as a marker of activation of the JNK/AP-1 pathway, is induced in brain tissue in the endovascular puncture model [35]. Treatment with Ac-YVAD-CMK treatment, a caspase-1 inhibitor, reduces JNK phosphorylation, leading to downregulation of the pro-inflammatory response [35]. Inhibition of JNK by SP600125, an ATP-competitive JNK inhibitor, results in neuroprotection measured as a decrease of cell death as shown by TUNEL staining and decreased cleaved caspase-3, reduced edema formation and BBB leakage, reduced MMP9 and improved functional outcome in the endovascular puncture model [109]. Additionally, in the blood injection model of SAH, it has also been shown that SP600125 can reduce caspase-3 activation, vasospasm, infiltration of leukocytes and IL-6 production [110,111].

More recently, a specific small peptide inhibitor of the JNK pathway, D-JNKi, has been shown to be neuroprotective in several brain damage models [112-116]. D-JNKi prevents interaction of JNK with its downstream and upstream targets, like the AP-1 transcription factor and MKK4/7 respectively. Moreover, D-JNKi was shown to strongly inhibit activation of mitochondrial JNK, which was identified as an important first step in setting off the apoptotic cascade [117]. Neuroprotection by D-JNKi in several models of brain injury is strongly associated with inhibition of pro-inflammatory cytokine/chemokine expression, inhibition of apoptotic cell death, reduction of brain injury and improvement of motor and cognitive behavior (see Figure 1) [112-116]. These data together indicate that inhibition of the JNK pathway might lead to inhibition of both inflammatory and cell death pathways, which might greatly enhance the efficacy of treatment.

#### NF-κB pathway

Nuclear factor-kappa B (NF-κB) is another transcription factor that regulates gene expression involved in inflammation, cell survival and apoptosis. NF-κB can increase pro-apoptotic factors in neurons and glial NF-κB activation induces expression of pro-inflammatory cytokines [118,119]. NF-κB seems to be a key regulator in the formation of cerebral aneurysms, which are probably at the origin of SAH [120,121]. SAH increases NF-κB activation in the brain during SAH in the endovascular puncture model, which is associated with an increased production of several inflammatory mediators [54,61,121]. You *et al*. (2013) have shown in the double-hemorrhage model of SAH that inhibition of NF-κB by PDTC, a proteasome inhibitor, reduces TNF-α, IL-1β and ICAM mRNA expression after SAH [121]. A more specific NF-κB inhibitor could be TAT-NBD, a small peptide inhibitor of the inhibitory kappa B kinase (IKK)/NF-κB pathway. TAT-NBD has been shown to strongly reduce neonatal brain injury, reduce cytokine/chemokine expression and improve long-term motor and cognitive behavioral outcome [122,123]. Moreover, we have shown previously that TAT-NBD strongly reduced apoptotic cell death, that is, caspase-3 activation and cytochrome *c* leakage, after ischemic brain injury [122,123].

Of interest, the possible effect of NF-κB inhibition and thereby the reduction of inflammation in SAH has also been shown by Suzuki *et al*. using recombinant-osteopontin (r-OPN) an endogenous extracellular matrix glycoprotein which is actively secreted after brain injury [61,124]. Besides NF-κB inhibition, OPN acts as a growth factor, increasing neural progenitor cell (NPC) proliferation and differentiation, allowing r-OPN to be a theoretically suitable therapy [125]. Suzuki *et al*. (2010) showed that r-OPN reduced NF-κB activation and MMP-9 expression in the endovascular puncture model [61]. Furthermore, r-OPN treatment improved the neurological score and decreased BBB permeability after SAH. In general, an increase in permeability of the BBB contributes to inflammation and neuronal damage, since it facilitates the trafficking of inflammatory cells and mediators across the BBB. MMP-9 favors BBB permeability by degrading the ECM. However, although the neurological score was improved early after SAH after r-OPN treatment, there was no significant difference in mortality between vehicle-treated animals and r-OPN-treated animals. Important to note is that besides the role of NF-κB in damaging pathways, NF-κB can also promote survival in neurons by the increase of anti-oxidants and anti-apoptotic molecules [119]. Thereby, NF-κB can have a dual role in brain damage and inhibition of NF-κB could reduce neuronal survival indicating that besides inhibiting inflammation, a more pronounced inhibition of cell death might be necessary to effectively decrease brain damage and mortality [61]. Since the NF-κB pathway is a classic example of a regulatory pathway of both inflammation and cell death, inhibition of this pathway by a specific inhibitor like TAT-NBD might be promising (see Figure 1).

### Possible neuroregenerative strategies

An important drawback of neuroprotective interventions is the relatively short therapeutic window. Therefore, therapies to repair the already damaged brain would be an interesting goal for treatment of SAH patients.

Erythropoietin (EPO), a sialoglycoprotein, has recently been suggested as a possible therapeutic option for SAH [126-128]. EPO has been shown to reduce brain injury in various types of cerebral insults [128-132]. EPO enhances neurogenesis as a neuronal growth factor, but EPO can also counteract inflammation and apoptosis [133-137]. With respect to SAH, Grasso *et al*. (2002) and Cheng *et al*. (2009) showed that treatment with rhEPO improves neurological outcome and decreases cell death after SAH [138,139]. To our knowledge, rhEPO has not been tested in the endovascular puncture model. Interestingly, Helbok *et al*. (2012) showed in a pilot study that EPO increases cerebral blood flow and oxygen delivery to the brain after SAH in humans [127]. Especially in SAH patients with a severe lesion, EPO is suggested to have beneficial effects on outcome [127]. These data indicate that EPO might become a safe and realistic option for treatment of patients with SAH, but the robustness of the therapy still has to be proven in a larger study (Figure 1).

Another potential neuroregenerative strategy could be the use of stem cell transplantation. Mesenchymal stem cells (MSCs) are known to interact with many cell types in the brain, like neural stem cells, cerebral endothelial cells, astrocytes and neurons, thereby boosting endogenous neurogenesis, repair and axonal sprouting [140]. In several brain damage models, mortality and lesion size were decreased after MSC transplantation [140-146]. In mice, MSCs can be administered as late as ten days after induction of neonatal brain damage, underpinning the long time-window of MSC transplantation [147]. Another advantage is that the intranasal route can be used for MSC administration allowing a rapid, and effective route of administration [144,147]. Khalili *et al*. (2012) showed in a SAH model of autologous blood injection that MSC transplantation at 24 hours after SAH improves neurological function [148]. Preliminary experiments in our group indicate that MSC also improve neurological functioning in the endovascular puncture model, suggesting that MSC transplantation may become a promising therapy for SAH with a long time window (Figure 1) [146,147].

It is of importance to note that strategies aiming at inhibition of pro-inflammatory cytokine expression, including those described above, could potentially result in reduced neuroregeneration [149,150]. Synthesis of cytokines and growth factors is necessary for proliferation and differentiation of neural precursor cells (NPC) [149,150]. Moreover, inflammatory cells are responsible for migration of neuronal precursor cells towards the side of damage by the expression of cytokines and chemokines.

### Towards clinical application

The studies reviewed here indicate that inflammation, as well as activation of multiple cell death pathways are important contributors to development of SAH brain injury. In this paper we review a hypothesis-driven selection of strategies that could potentially be used to combat SAH brain damage. We have focused on the endovascular puncture model as this animal model closely mimics SAH pathology in humans. The currently known inflammatory responses and cell death mechanisms that we described to be operative in the endovascular model of SAH are to a large extent also operative in other experimental models of SAH.

The actual clinical translation of the therapeutic strategies described in this review will be most effective when the drugs allow administration with a therapeutic window of at least a few hours. Moreover, as brain injury after SAH develops as a result of an interplay of several detrimental pathways, including inflammation and multiple routes of cell death, we suggest that neuroprotective inhibitors that target both inflammation and cell death, like D-JNKi or TAT-NBD, are promising candidates to combat SAH brain injury. When targeting cell death specifically, a strategy targeting the upstream in the apoptotic cascade, for example, PFT-μ, D-JNKi or BIP, would be preferable. These drugs inhibit damage to the mitochondrial outer membrane, thereby preventing activation of the downstream cascade, which is a more realistic option than targeting one specific caspase in the cascade. In this respect, it is important to realize that several forms of cell death might be operative in the SAH injured brain, so the use of necrostatin-1, which has been shown to inhibit multiple cell death pathways might also be a promising candidate.

Finally, we would like to underline the importance of neuroregenerative strategies like intranasal MSC administration to repair cerebral injury induced by SAH, especially when treatment cannot be started within a few hours after the insult. Moreover, regenerative strategies could also be considered as an add-on therapy of neuroprotective strategies for enhancing tissue repair.

## Abbreviations

AP-1: Activating protein-1; BBB: Blood brain barrier; BIP: Bax-inhibiting peptide; CBF: Cerebral blood flow; CPP: Cerebral perfusion pressure; CSF: Cerebrospinal fluid; DC: Decompressive craniectomy; DCI: Delayed cerebral ischemia; ECA: External carotid artery; ECM: Extracellular matrix; EPO: Erythropoietin; ERK: Extracellular signal-regulated kinases; FDA: Food and Drug Administration; GA: Glycyrrhizic acid; HMGB1: High mobility group box 1; HSP70: Heat shock protein 70; ICAM-1: Intercellular adhesion molecule-1; ICP: Intracranial pressure; IκB: Inhibitory kappa B; IKK: IκB kinase; IL: Interleukin; JNK: c-Jun N-terminal kinase; MABP: Mean arterial blood pressure; MAP: Mitogen activated protein; MCA: Middle cerebral artery; MMP9: Matrix metalloproteinase 9; MRI: Magnetic resonance imaging; MSC: Mesenchymal stem cell; NBD: NEMO binding domain; NF-κB: Nuclear factor-kappa B; NO: Nitric oxide; NPC: Neural progenitor cell; OPN: Osteopontin; PFT: Pifithrin; r-OPN: recombinant osteopontin; RAGE: Receptor for advanced glycation end products; rhEPO: recombinant human erythropoietin; ROS: Reactive oxygen species; SAH: Subarachnoid hemorrhage; TLR: Toll like receptor; TNF: Tumor necrosis factor; TUNEL: Terminal deoxynucleotidyl transferase dUTP nick end labeling; VCAM-1: Vascular cell adhesion molecule-1.

## Competing interests

The authors declare that they have no competing interests.

## Authors’ contributions

EK identified articles, drafted and edited the manuscript and drafted the figure. CHN co-wrote the manuscript and assisted in the planning and editing of the manuscript. CJH proposed the scope of the review and assisted in the planning and editing of the manuscript. CTJV, AK and JK assisted in the planning and editing of the manuscript. All authors read and approved the final manuscript.

## Funding

This study was funded in part by the Dirkzwager-Assink foundation and by the Friends of University Medical Center Utrecht foundation.
